# Characterisation of Pea Milk Analogues Using Different Production Techniques

**DOI:** 10.17113/ftb.62.02.24.8356

**Published:** 2024-06

**Authors:** Ali Emre Andaç, Necati Barış Tuncel, Neşe Yılmaz Tuncel

**Affiliations:** 1 Onsekiz Mart University, Faculty of Engineering, Department of Food Engineering, 17100 Çanakkale, Turkey; 2 Onsekiz Mart University, Faculty of Applied Sciences, Department of Food Technology, 17100 Çanakkale, Turkey

**Keywords:** plant-based milk analogues, plant-based milk substitutes, off-flavour, lipoxygenase, vacuum, gas chromatography-olfactometry

## Abstract

**Research background:**

Among legumes, peas are characterised by their high protein content, low glycaemic index and exceptional versatility. However, their potential as a food is often compromised by their undesirable off-flavour and taste. Hence, this study focuses on minimising off-flavours through simple pretreatments with the aim of improving the potential for the production of pea milk analogues. Pea milk analogues are a burgeoning type of plant-based milk alternatives in the growing plant-based market.

**Experimental approach:**

Pea seeds were subjected to different pretreatments: (*i*) dry milling, (*ii*) blanching followed by soaking in alkaline solution and subsequent dehulling and (*iii*) vacuum. Typical physicochemical properties such as pH, viscosity, colour, titratable acidity and yield were measured to obtain a brief overview of the products. Consumer acceptance test, descriptive sensory analysis, gas chromatography-mass spectrometry and gas chromatography-olfactometry were used to map the complete sensory profile and appeal of the pea milk substitutes.

**Results and conclusions:**

The L* values of the pea milk analogues were significantly lower than those of cow’s milk, while a*, b*, viscosity and pH were similar. In the descriptive sensory analysis, sweet, astringent, pea-like, cooked, hay-like, boiled corn and green notes received relatively higher scores. The vacuum-treated pea milk analogues received higher scores for flavour and overall acceptability in the consumer acceptance test. The pretreatments resulted in significant changes in the volatile profiles of the pea milk analogues. Some volatiles typically associated with off-flavour, such as hexanal, were found in higher concentrations in blanched pea milk analogues. Among the applied pretreatments, vacuum proved to be the most effective method to reduce the content of volatile off-flavour compounds.

**Novelty and scientific contribution:**

This study stands out as a rare investigation to characterise pea milk analogues and to evaluate the impact of simple pretreatments on the improvement of their sensory properties. The results of this study could contribute to the development of milk alternatives that offer both high nutritional value and strong appeal to consumers.

## INTRODUCTION

In recent years, consumers have reduced their consumption of animal products due to growing awareness of sustainability, environmental impact of food and concerns about diseases associated with animal-based diets (*1*). In response to these trends, food manufacturers and researchers are developing plant-based alternatives such as meat and dairy analogues. The plant-based food market, open for further expansion and innovation, has experienced rapid growth in recent years and is expected to reach USD 161.9 billion by 2030 (*2*). Plant-based milk analogues represent the largest product category of the plant-based market (*3*). Plant-based milk analogues are water-soluble extracts of plant materials and they are similar in appearance and consistency to cow’s milk.

Pulses are considered the most important raw materials for plant-based milk analogues due to their protein-rich and nutrient-rich properties. Commercially, the most popular and accessible pulse-based milk analogue is soy milk (*4*). Soybean is one of the richest sources of protein among pulses. However, soy allergy restricts the consumption of soy products (*5*). In addition, antinutrients such as enzyme inhibitors and tannins reduce the bioavailability of soy protein (*6*).

Peas, soybeans, wheat and rice are the most important sources for the production of plant-based alternatives (*7*). Peas are becoming a promising alternative to soy for the production of plant-based milk analogues due to their low allergenicity, widespread availability, and high nutritional value, thus attracting more and more attention (*8*). Pea (*Pisum sativum* L.) is one of the oldest crops in the world and is grown in 84 countries, including Australia, Canada, China and the United States (*9*). Moreover, the pea has the largest share (36 %) of total pulse production worldwide (*10*). Therefore, it is recognised as an excellent source of nutrients, especially its high-quality protein. Pea protein (~20–25 % of pea seed) is rich in essential amino acids such as tryptophan and lysine and characterised by its high digestibility and notably fewer allergenic reactions than soybean or other plant proteins (*10*). Peas are also rich in soluble and insoluble fibre, low in fat and sodium and a remarkable source of complex carbohydrates, B-group vitamins, folate and minerals, especially iron, calcium and potassium (*9*). In addition, the consumption of peas is associated with various health benefits, such as anticancer, antiobesity, antidiabetic and cardioprotective effects (*11*). However, the use of peas in food is limited, partly due to their undesirable sensory attributes, known as ’beany off-flavour‘ (*12*).

The off-flavour of peas can either be inherent or develop during processing and storage (*13*). The main off-flavours in peas are described as green, beany, earthy, hay-like, bitter and astringent. They are associated with volatile compounds such as aldehydes, ketones and alcohols, as well as non-volatile compounds such as isoflavones and saponins (*13*, *14*). The presence of off-flavour related volatiles is mostly attributed to the oxidation of unsaturated fatty acids catalysed by enzymes (*15*). In this context, lipoxygenase (LOX), hydroperoxide lyase enzymes and indirectly lipase have been reported to play an important role in the formation of volatile off-flavour compounds (*16*, *17*).

There are only a few studies on the improvement of the sensory properties of products made from green pea seeds. Azarnia *et al*. (*18*) investigated the volatiles in yellow, green and greyish-brown cotyledons of field pea cultivars grown under uniform conditions to evaluate the effect of cultivar, harvest year and processing methods (dry milling, cooking and dehulling) on the volatile flavour compounds. The authors indicated that the volatile flavour compounds in peas were affected by the cultivar, harvest year and processing conditions. Moreover, cooking significantly reduced the total area counts of these volatile compounds.

Bi *et al*. (*19*) performed roasting (160 °C for 30 min), high hydrostatic pressure (200–550 MPa for 10 min) and treatment with inhibitors (ascorbic acid, quercetin, epigallocatechin-3-gallate and reduced glutathione) to improve the sensory properties of pea milk. The authors found that high hydrostatic pressure in combination with quercetin had the best inhibitory effect on LOX-2 enzyme activity, which correlated significantly with hexanal content.

Ma *et al*. (*8*) applied different pretreatments to dried yellow peas, such as dehulling, blanching, acid soaking, alkaline soaking and their combinations. The authors produced pea milk yoghurt and found that a combination of blanching and acid soaking led to the highest sensory scores, as evaluated by a panel of ten trained members. It was concluded that this pretreatment improved the sensory appeal compared to the control sample. Yen and Pratap-Singh (*15*) reported that microwave-vacuum drying significantly reduced the total volatile compounds in pea protein and had great potential to reduce off-flavour intensity. Lan *et al*. (*20*) evaluated the effects of spray drying based on solid dispersions on the sensory properties of pea protein isolate and found that dispersions with gum Arabic and maltodextrin reduced the beany flavour. Tanger *et al*. (*21*) reported that both spray drying and freeze-drying reduced the beany off-flavour and improved the sensory properties of pea protein.

The main objective of this study is to evaluate the effectiveness of simple pretreatments (dry milling, which served as a control, blanching followed by soaking in alkaline water and subsequent dehulling, and vacuum), which can be easily transferred to large-scale production, in mitigating the characteristic off-flavour in pea milk analogues and to investigate the correlation between LOX activity and sensory acceptance. A further aim is to investigate the effect of the treatments on the physicochemical and sensory properties of pea milk analogues.

## MATERIALS AND METHODS

### Materials

Pea (*Pisum sativum* L.) seeds were purchased at local markets in Çanakkale, Turkey. Pea seeds from three different brands were combined to increase the representativeness of the sample. The combined material had average mass fraction of moisture, crude protein, ash, crude fat, insoluble, soluble and total dietary fibre of 9.25, 24.05, 2.83, 2.32, 7.97, 0.56 and 8.53 %, respectively. The moisture mass fraction was measured at 130 °C (*22*). The crude protein mass fraction was measured by the macro-Kjeldahl method, with a nitrogen conversion factor of 6.25 to calculate the protein content (*23*). The ash mass fraction of the samples was measured by linear heating to 650 °C (*24*). The crude fat mass fraction was determined by the Soxhlet method with hexane as the solvent (*25*). Soluble, insoluble and total dietary fibre mass fractions were analysed using a commercial enzyme kit (Megazyme, Wicklow, Ireland) by an enzymatic-gravimetric mechanism (*26*).

Additionally, three different brands of whole milk and two different brands of semi-skimmed cow's milk were purchased to compare some physicochemical properties.

### Pea seed pretreatments

Three different pretreatments of pea seeds were used: (*i*) dry milling (control): pea seeds were ground with a laboratory grinder (IC-02A; Yuhong Industry, Jiangsu, PR China) and sieved through a 300-μm sieve, (*ii*) blanching followed by soaking in alkaline solution and then dehulling. The pea seeds were blanched by immersing them in boiling water (~100 °C) for 3 min to inactivate the LOX enzyme. They were then soaked in alkaline water (pH=9) for 1 h, dehulled manually and wet milled using a blender (8011S; Waring, Stamford, CT, USA) for 5 min at high speed, and (*iii*) vacuum: the pea seeds were dry milled and then hydrated for 30 min on a magnetic stirrer at room temperature. The suspension (*m*(solid):*V*(water)=1:10) was then transferred to a rotary evaporator (RV 8; IKA, Staufen, Germany) and subjected to a constant vacuum (0.08 MPa) at 50 °C for 30 min with a rotation speed of 50 rpm. The pea milk analogues produced from peas subjected to the above pretreatments were named DPMA, BPMA and VPMA.

### Determination of LOX activity

The LOX activity of pea seeds was determined according to Lampi *et al*. (*27*) with some modifications. To extract LOX, 10 g of pea seeds were weighed and milled with distilled water (1:10) in a blender (8011S; Waring) for 2 min. The mixture was centrifuged (NF 800R; Nüve, Ankara, Turkey) at 9435×*g* and 4 °C for 15 min and the supernatant was used as enzyme extract after dilution with M/15 phosphate buffer, pH=6.8. The substrate was a 10 mM linoleic acid (Sigma-Aldrich, Merck, St. Louis, MO, USA) solution in 1 % Tween 20 in water, which was clarified with 1 M NaOH. The change in absorbance at 234 nm was recorded immediately (UV-160A; Shimadzu, Kyoto, Japan) after the addition of 0.2 mL of enzyme extract to a mixture of 2.6 mL of M/15 phosphate buffer and 0.2 mL of substrate solution for a period of 270 s. The LOX activity results were calculated using the following equation proposed by Baltierra-Trejo *et al.* (*28*):


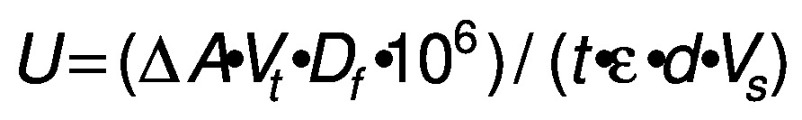
 /1/

where U is the enzyme activity (μmol/(min·L)), Δ*A* is the difference between the final and initial absorbance, *V*_t_ is the total reaction volume (mL), *D*_f_ is the dilution factor, 10^6^ is the concentration correction factor (μmol/mol), *t* is the reaction time (min), *ε* is the molar absorption coefficient (26 000 M^-1^·cm^-1^), *d* is the optical path (1 cm) and *V*_s_ is the final volume of the sample (mL).

### Production of pea milk analogues

All samples of pea milk analogues were prepared at *m*(pea):*V*(water)=1:10 for comparison. The suspension was exposed to the above pretreatments, then filtered through <100 µm sieve and heated at about 80 °C for starch gelatinisation. The starch was hydrolysed with commercial α-amylase enzyme (LT-300; Spezyme, Dupont, DE, USA) according to the instructions (1 µL enzyme solution per g sample). The mixture was then homogenised (T25 Digital; IKA) at 3276×*g* for 5 min and was sterilised in a screw-capped glass bottle (1 L) at 121.1 °C for 5 min using autoclave (HV-110L; Hirayama, Tokyo, Japan).

### Physicochemical analysis

The viscosity of the final pea milk analogues (after sterilisation) was measured at 20 °C using a viscometer (LVDV-II+Pro; Brookfield, Toronto, Canada) equipped with an SC4-18 spindle rotating at a shear rate of 264 s^-1^. The colour of the final pea milk analogues was measured according to CIE L*a*b* system using a cylindrical cuvette (cell holder CR-A503, tube cell CR-A504; Minolta, Osaka, Japan) and a colorimeter (CR-400; Minolta). Whiteness was calculated according to Milovanovic *et al.* (*29*). A digital pH meter (S20; Mettler Toledo, Colombus, OH, USA) was used for pH measurements. Titratable acidity was determined according to Nielsen (*30*) and the results were expressed as mass fraction of lactic acid equivalents in %. The yield was determined according to Moscoso Ospina *et al*. (*31*) and calculated as a mass fraction of sterilised pea milk analogue in its initial wet mass.

### Consumer acceptance test

The effect of the pretreatments on the sensory appeal of the pea milk analogues was evaluated using a consumer acceptance test according to Meilgaard *et al*. (*32*). The participants (approx. 60 % female and 40 % male) were predominantly university staff and students (*N*=58) aged from 21 to 53. A 9-point hedonic scale (1=dislike extremely, 2=dislike, 3=dislike moderately, 4=dislike slightly, 5=neither like nor dislike, 6=like slightly, 7=like moderately, 8=like, 9=like extremely) was used for the evaluation. The samples of pea milk analogues were coded with random three-digit numbers and served to panellists in plastic cups (~20 mL) at room temperature and under daylight. Drinking water was served between samples to cleanse the palate.

### Descriptive sensory analysis

The sensory attributes of the pea milk analogues were evaluated using a descriptive sensory analysis according to Meilgaard *et al*. (*32*). Seven trained panellists (5 females, 2 males) aged between 27 and 54 developed potential sensory terms by tasting different types of commercial plant-based milk analogues in several rounds. The definitions and references of the developed descriptive terms are given in Table 1. Each type of milk analogue was assessed in duplicate for the sensory attributes using a 15-point scale (0 represents no attribute and 15 indicates a strong presence of the attribute). The samples of pea milk analogues were coded with random three-digit numbers and served to panellists in plastic cups (~30 mL) at room temperature. Unsalted crackers and drinking water were provided between samples to cleanse the palate.

**Table 1:** Definitions and references for the descriptive terms used in descriptive sensory analysis

Sensory term	Description	Reference
Sweet	Taste sensation elicited by sugars	*w(*(sucrose)_aq_=2 %, HS=2.0*
		*w*(sucrose)_aq_=5 %, HS=5.0
Salty	Taste sensation elicited by salts	*w*(NaCl)_aq_=0.2 %, HS=2.5
		*w*(NaCl)_aq_=0.35 %, HS=5.0
Bitter	Taste sensation elicited by caffeine	*w*(caffeine)_aq_=0.05 %, HS=2.0
		*w*(caffeine)_aq_=0.08 %, HS=5.0
Sour	Taste sensation elicited by citric acid	*w*(citric acid)_aq_=0.05 %, HS=2.0
Umami	Taste sensation elicited by certain amino acids (glutamate and aspartate) and nucleotides	*w*(monosodium glutamate)_aq_=0.5 %,HS=3.0
		*w*(monosodium glutamate)_aq_=0.75 %, HS=4.5
Astringent	The shrinking or puckering of the tongue surface caused by substances such as tannins or alum	Tea (brewed)
Pea-like	Aromatics associated with pea	Pea (boiled)
Cooked	Aromatics associated with cooked cereals and pulses	Bulgur (boiled)
Sulphurous	Aromatics associated with sulphurous compounds	Egg (boiled)
Nutty	Aromatics associated with hazelnut/peanut	Hazelnut/peanut (crushed)
Earthy	Aromatic notes associated with damp soil, wet foliage or slightly undercooked potatoes	Green potato skin
Hay-like	Aromatics associated with neutral notes	Oats (soaked)
Boiled corn	Aromatics associated with boiled sweet corn	Canned sweet corn
Polish	Aromatics associated with polish	Flaxseed (oxidised)
Dirty wet towel	Aromatics associated with dirty and wet towel	Reference not used/assignment by panellists
Metallic	Aroma of minerals and metals commonly associated with metal spoon	Reference not used/assignment by panellists
Green/flower	Aromatics associated with freshly cut leaves, grass and unripe fruits	Freshly cut green grass
Fermented dough	Aromatics associated with fermented dough	Dough (fermented)
Medicinal	Aromatics associated with medicine	Crushed vitamin B complex
Wet cardboard	Aromatics associated with wet cardboard	Wet cardboard

*Reference numbers for the basic taste indicate their position on the 15-point hedonic scale (HS)

### Gas chromatography-mass spectrometry analysis

The volatile compounds of pea milk analogues were extracted with the headspace solid-phase microextraction (HS-SPME) method and identified with gas chromatography-mass spectrometry (GC-MS). Briefly, 5 mL of sample, 1 g of NaCl and 10 μL of internal standard (10 μL of 2-methyl-3-heptanone in 5 mL methanol) were mixed in a 40-mL amber vial capped with a PTFE/silicone septum (Supelco, Bellefonte, PA, USA). The content was incubated in a water bath at 50 °C for 30 min. Then, SPME fibre (Carboxen/DVB/PDMS 50/30 μm 2 cm; Supelco) was inserted into the vial and incubated under the same conditions for another 30 min to absorb volatile compounds. At the end of that period, SPME fibre was injected into the GC-MS (HP 6890 GC and 7895C mass selective detector; Agilent, Santa Clara, CA, USA) in splitless mode. HP-INNOwax column (60 m×0.25 mm i.d., 0.25 μm film thickness; J&W Scientific, Agilent) was used for the separation of volatile compounds. Helium was used as carrier gas at a flow rate of 1 mL/min. The GC oven temperature was initially set at 40 °C for 1 min, then ramped up to 250 °C at a rate of 4 °C per min, with a final hold time of 10 min. The MS was operated at ionization energy of 70 eV, interface temperature of 280 °C, mass range from 35 to 350 *m*/*z* and scan rate of 4.45 scan**/**s. National Institute of Standards and Technology (NIST) (*33*) and Wiley Registry of Mass Spectral Data libraries (*34*) were used for the identification of volatile compounds (based on >70 match score). Retention indices were calculated according to Van den Dool and Kratz (*35*) using *n*-alkane series (C_7_-C_23_) (Sigma-Aldrich, Merck) as external references.

### Gas chromatography-olfactometry analysis

Aroma-active compounds of pea milk analogues were extracted with the HS-SPME as mentioned above with the exception of the addition of internal standard. The SPME fibre equipped with an olfactory detection port was then injected into the GC system (HP 6890 GC; Agilent). DB-5 column (30 m×0,32 mm i.d., 0,25 μm film thickness; J&W Scientific, Agilent) was used for the identification of aroma-active compounds. Helium with a flow rate of 1.7 mL/min was used as a carrier gas. The GC oven temperature was initially set at 40 °C for 3 min, then ramped up to 200 °C at a rate of 10 °C per min, with a final hold time of 10 min. Intensities of aroma-active compounds were determined with a 10-point scale (left side: 0=no intensity, right side; 10=strong intensity). Odour descriptions were compared with: (*i*) *n*-alkane series (C_7_-C_23_) (Sigma-Aldrich, Merck), which were injected under the same chromatographic conditions and the retention indices of each compound were matched to the NIST database (*33*) and literature, (*ii*) data obtained with GC-MS, and (*iii*) authentic standard compounds which were analysed under the same chromatographic conditions.

### Statistical analysis

The data were evaluated using Minitab v. 21.4.2 (*36*), SPSS v. 27.0.1.0 (*37*) and NCSS v. 11 (*38*) statistical software. Parametric data were assessed with analysis of variance (one-way ANOVA) and multiple comparisons were made with Tukey’s test (p<0.05). Non-parametric data were assessed with the Kruskal-Wallis test and multiple comparisons were made with Dunn’s test (p<0.05). All data were expressed as mean value±standard error. The mean values are of three replicates except for the GC-O analyses, which were conducted twice.

## RESULTS AND DISCUSSION

### LOX activity

**Fig. 1:**
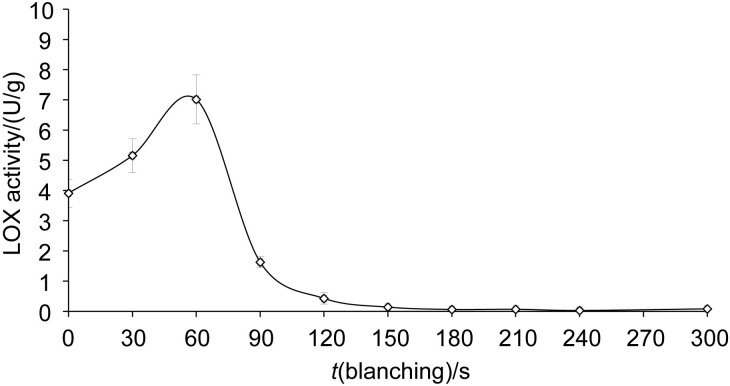
Lipoxygenase (LOX) activity as a function of the blanching time

It is widely acknowledged that the volatile compounds responsible for inducing off-flavours primarily result from LOX enzyme activity, which catalyses the oxidation of unsaturated fatty acids in the presence of oxygen (*17*). Additionally, the LOX enzyme is associated with quality loss as it leads to discolouration, pigment degradation and loss of essential fatty acids (*16*). In this regard, the inactivation of the LOX enzyme appears to be crucial for pea processing. The effect of blanching on LOX activity as a function of process time is shown in Fig. 1. It was determined that LOX was completely inactivated after 3 min of blanching. In addition, it was observed that LOX activity increased in the early stages (0-60 s) of blanching and after that it showed a decreasing trend (Fig. 1). This is most likely due to inhomogeneous heat transfer. In other words, different regions of the grain reached the temperature at which the enzyme is inactivated at different times. Similar results were found by Gökmen *et al*. (*39*), who reported complete inactivation after blanching at 80 °C for 2 min.

### Physicochemical properties of pea milk analogues

The physicochemical properties of the pea milk analogues are shown in Table 2. Viscosity is a critical physical parameter used in quality control related to mouthfeel. During the preliminary assessments, it was observed that the viscosity of the pea milk analogues was primarily correlated with the mass fraction of solids and the hydrolysis of starch. It was not possible to obtain a final product with a drinkable viscosity after sterilisation if starch hydrolysis was not performed. The viscosity of the pea milk analogues, which were prepared at the same solid mass fraction (10 %), ranged between 2.53 and 3.25 mPa·s. The viscosity of both whole and semi-skimmed cow's milk samples from various brands, measured using the same method, ranged between 1.9 and 2.1 mPa·s. Similar viscosities for semi-skimmed (1.56 mPa·s) and whole cow’s milk (2.00 mPa·s) were reported by Nikmaram and Keener (*40*). Jeske *et al.* (*41*) evaluated the physicochemical properties of 17 commercial plant-based milk analogues and found that their viscosity varied widely between 2.21 and 47.80 mPa·s. It is worth mentioning that the viscosity of the final product can be significantly modified by the hydrolysis of raw materials with high content of starch.

**Table 2:** Physicochemical properties of the pea milk analogues

Property	DPMA	BPMA	VPMA
*η*/(mPa·s)	(2.97±0.07)^b^	(2.53±0.03)^c^	(3.25±0.03)^a^
pH	(6.86±0.01)^a^	(6.85±0.01)^ab^	(6.84±0.01)^b^
TA as *w*(lactic acid)/%	(0.08±0.01)^a^	(0.06±0.01)^b^	(0.08±0.01)^a^
*Y*/%	(87.20±1.75)^a^	(72.21±1.60)^c^	(83.22±1.90)^b^
L*	(44.46±0.03)^b^	(43.46±0.01)^c^	(47.89±0.02)^a^
a*	(-4.04±0.01)^c^	(-2.98±0.01)^a^	(-3.93±0.01)^b^
b*	(5.05±0.01)^b^	(4.77±0.01)^c^	(6.47±0.01)^a^
Whiteness	(44.08±0.03)^b^	(43.18±0.01)^c^	(47.35±0.02)^a^

Results are expressed as mean value±standard error. Mean values followed by different letters in superscript within the same row are significantly different (p<0.05). DPMA, BPMA and VPMA=pea milk analogues pretreated with dry milling, blanching and vacuum, respectively, TA=titratable acidity

The pH and titratable acidity expressed as lactic acid of unformulated pea milk analogue were in the range of 6.84–6.86 and 0.06–0.08 %, respectively (Table 2). Similar pH and titratable acidity values were reported in other studies about plant-based milk analogues (*42*). On average, the pH and titratable acidity expressed as lactic acid of commercial cow’s milk samples were 6.5 and 0.16 %, respectively.

The yield of the pea milk analogues ranged between 72.2 and 87.2 %, with dry milling resulting in a significantly higher yield than wet milling (p<0.05) (Table 2). Previous studies have reported much lower yield values, in the range 50–60 % (*43*). The difference in yield values may be attributed to different processes, particularly the filtration and milling of the raw material, as well as differences in calculation methods.

Colour is a sensory attribute that significantly affects consumer preference. L* and whiteness values of pea milk analogues were quite low compared to cow’s milk. The L* value of pea milk analogue ranged between 43.46 and 47.89 (Table 2), while the L* value of commercial cow's milk samples was between 76 and 79 (data not shown). The darker colour of the pea milk analogue was attributed to the chlorophyll degradation and non-enzymatic browning reactions that may occur during sterilisation. Similarly, studies have reported that the colour of soy milk that was heat-treated at increased temperatures is adversely affected by Maillard reactions. Additionally, the browning index of soy milk has been observed to increase with longer holding times at high temperatures (*44*). The lower L* value in BPMA, which involves a dehulling step, suggests that the pigments are not concentrated in the hulls of peas, unlike other pulses such as lentils, faba beans and mung beans (*45*). It is also important to note that ingredients added during the formulation step of PBMA can have a significant effect on the colour of the final product. For instance, the addition of oil and homogenisation of the mixture can result in a significant increase in the L* value (data not shown). The calculated whiteness value followed exactly the same trend as L* value (Table 2). Negative a* values, indicating greenness, and positive b* values, indicating yellowness, were observed in this study (Table 2) and the results were similar to those of the commercial cow’s milk samples. On the other hand, Oliveira *et al.* (*46*) reported a decrease in L* and an increase in a* and b* values when increasing concentrations of pea protein isolate were added to skimmed cow’s milk.

### Consumer acceptance of pea milk analogue

The results of the consumer acceptance test for pea milk analogues are shown in Table 3. In consumer acceptance tests, food products are usually presented in their final form in which they would be consumed. However, the pea milk analogues were produced and presented in unformulated form to eliminate the masking effect of ingredients such as sugar and flavourings. Therefore, it is important to emphasise that these results apply to unformulated samples. Additionally, the addition of ingredients, especially sugar during the formulation stage, significantly increases consumer acceptance. Despite being unformulated, all samples received scores above 5 (meaning neither like nor dislike) on a 9-point hedonic scale (Table 3). The participants could not detect any significant difference between the samples subjected to different pretreatments in terms of appearance and consistency (p>0.05). However, VPMA received the highest scores for aroma/flavour and overall acceptability, which can be attributed to the volatilisation of undesirable off-flavours in a water bath at 50 °C and their subsequent elimination under vacuum. The vacuum treatment was carried out on a laboratory scale, suggesting that more efficient results can be achieved with vacuum systems on an industrial scale. Vacuum treatment has also been described as an effective strategy for removing beany flavour from soy milk (*47*). While no statistically significant difference was found between the consumer scores, DPMA received the lowest overall acceptance score on average, which was very close to that of BPMA (Table 3). Therefore, it can be hypothesised that blanching and dehulling pretreatments did not have a positive effect on the overall sensory perception of the pea milk analogue. In other words, the inactivation of LOX did not provide any additional benefit in terms of increasing consumer appeal. Similarly, Murat *et al.* (*48*) reported that off-flavours can occur even when LOX is inactivated. On the other hand, it is also worth noting that consumer acceptance tests are highly subjective and may not be reproducible when applied to a different or much larger consumer community.

**Table 3:** Consumer acceptance test results of the pea milk analogues

PMA type	Appearance*	Consistency*	Aroma/flavour*	Overall acceptability*
DPMA	(5.6±0.3)	(6.2±0.3)	(5.2±0.4)	(5.7±0.3)
BPMA	(5.7±0.4)	(6.2±0.3)	(5.6±0.3)	(5.8±0.3)
VPMA	(5.5±0.4)	(6.2±0.3)	(6.1±0.3)	(6.2±0.3)

Results are expressed as mean value±standard error. *The effect of the treatments is not significant (p>0.05). A 9-point hedonic scale was used, where 1=dislike extremely, 2=dislike, 3=dislike moderately, 4=dislike slightly, 5=neither like nor dislike, 6=like slightly, 7=like moderately, 8=like, 9=like extremely. DPMA, BPMA and VPMA=pea milk analogues pretreated with dry milling, blanching and vacuum, respectively

### Descriptive sensory analysis of pea milk analogues

**Fig. 2:**
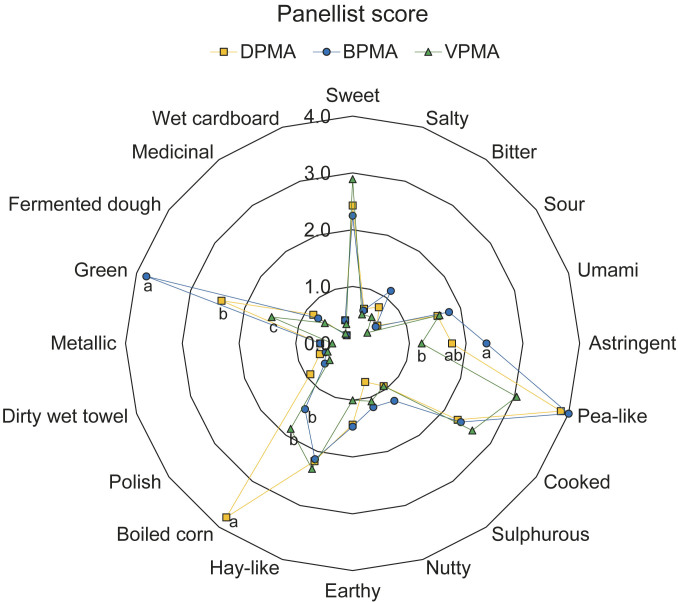
Descriptive sensory analysis results of the pea milk analogues. Values marked with different letters are significantly different (p<0.05). A 15-point hedonic scale was used, where 0 represents no attribute and 15 indicates a strong presence of the attribute. DPMA, BPMA and VPMA=pea milk analogues pretreated with dry milling, blanching and vacuum, respectively

The results of the descriptive sensory analysis of pea milk analogues are shown in Fig. 2. The panellists developed fifteen flavour descriptors, namely astringent, pea-like, cooked, sulphureous, nutty, earthy, hay-like, boiled corn, polish, dirty wet towel, metallic, green, fermented dough, medicinal and wet cardboard. Among these, sweet, astringent, pea-like, cooked, hay-like, boiled corn and green received relatively higher scores than the other descriptive terms (Fig. 2). Statistically significant differences were found in the scores of astringent, boiled corn, and green in relation to the pretreatments. Similar descriptive terms have been reported in previous studies on pea milk (*49*, *50*). Zhang *et al*. (*49*) found that “earthy” notes received the highest score in pea milk, followed by “grassy/green”, “mushroom” and “sweet”. Bi *et al.* (*19*) conducted a sensory evaluation of pea milk, in which trained panellists were instructed to list as many attributes as possible to describe the sensory profile. The researchers found that the five terms with the highest frequency among all defined attributes were raw beans, grassy, milk-like, earthy and fatty. Moreover, Trikusuma *et al*. (*50*) reported that the notes beany, potato, pasta and cooked green bean were the most frequent in pea protein beverage.

In the present study, it was found that vacuum pretreatment resulted to a significantly lower intensity of the notes astringent, boiled corn and green (p<0.05). In addition, the intensities of the sensory attributes pea-like, earthy, polish, dirty wet towel, metallic, fermented dough and wet cardboard were lower in VPMA (Fig. 2). The sensory descriptors mentioned above are primarily perceived as undesirable and are often associated with off-flavours. It can therefore be concluded that the results of the descriptive sensory analysis are consistent with those of the consumer acceptance test. On the other hand, the intensities of “pea-like” and “green” notes were the highest in BPMA, which underwent blanching pretreatment to inactivate LOX (Fig. 2). This finding suggests that the off-flavour of peas is not solely due to LOX enzyme activity, as has been emphasised by other researchers (*13*).

### GC-MS analysis of pea milk analogues

The volatile compounds of the pea milk analogues identified by GC-MS are listed in Table 4. Of the total 21 detected compounds, 9 of them - namely 2-ethyl-furan, 1-pentanal, hexanal, butanoic acid/2-methylpropyl ester, 2-heptanone, (*Z*)-2-heptenal, thujone, benzaldehyde and 2-furanmethanol - were present in all samples. The identified volatiles belong to different groups such as aldehydes, alcohols, ketones, esters, furans and phenols. Most of these identified volatiles are formed as a result of oxidation, enzymatic activity and/or Maillard reactions in materials such as pea flour, pea protein isolates and pea milk (*12*, *48*, *51*).

**Table 4:** Volatile profile of the pea milk analogue determined by gas chromatography-mass spectrometry analysis

					*γ*/(µg/L)	
Compound	RT	RI	Aroma description	DPMA	BPMA	VPMA
2-Ethyl-furan	5.92	944	Sweet, burnt	(4.1±0.8)^b^	(15.0±3.4)^ab^	(26.8±5.4)^a^
1-Pentanal	6.46	980	Almond, malt, pungent	(6.10±0.01)^b^	(11.4±1.9)^a^	(8.0±0.2)^ab^
Acetic acid butyl ester	7.93	1053	Pear	-	(0.83±0.03)^a^	(0.37±0.01)^b^
Hexanal	8.08	1060	Green	(47.4±3.3)^b^	(100.2±0.3)^a^	(43.05±4.02)^b^
1-Penten-3-ol	9.83	1131	Pungent	-	(8.5±3.6)	-
Butanoic acid, hexyl ester	9.85	1132	Green	(0.48±0.02)	-	-
Butanoic acid, 2-methylpropyl ester	9.89	1133	Fruity	(2.19±0.06)	(4.5±1.0)	(2.75±0.07)
2-Heptanone	10.96	1171	Soap	(12.4±1.9)	(56.4±17.9)	(19.3±2.0)
2-Pentyl-furan	11.65	1195	Green bean	-	(23.6±1.0)^ab^	(47.1±9.6)^a^
1-Pentanol	12.98	1232	Balsamic	-	(84.6±7.0)^a^	(4.5±1.8)^b^
1-Hexanol	17.11	1344	Resin, flower, green	(4.7±0.4)	-	(3.8±0.2)
(Z)-2-Heptenal	18.90	1394	Fish	(4.2±1.6)	(6.5±0.9)	(2.0±0.1)
Furfural	21.02	1459	Bread, almond, sweet	-	(2.3±0.3)	(1.91±0.01)
1-Octen-3-ol	21.61	1478	Mushroom	-	(4.5±0.2)	-
Thujone	22.30	1499	Thujonic	(5.9±0.3)	(7.26±0.09)	(11.1±2.7)
Benzaldehyde	23.53	1540	Almond, burnt sugar	(3.8±1.2)^ab^	(1.6±0.4)^b^	(6.3±0.7)^a^
1-Octanol	24.58	1575	Oily, aldehyde	-	-	(2.3±0.2)
2-Furanmethanol	25.08	1592	Burnt	(9.0±0.7)	(8.9±2.3)	(8.6±1.4)
Metoxyphenyl oxime	28.20	1732	Wet towel	-	-	(2.8±0.3)
α-Terpineol	28.84	1755	Oil, aniseed, mint	(5.5±0.2)	(5.0±0.5)	-
2-Metoxy-4-vinylphenol	39.37	2142	Clove, curry	(1.41±0.07)^a^	-	(0.57±0.03)^b^

Results are expressed as mean value±standard error, *N*=3. Mean values followed by different letters in superscript within the same row are significantly different (p<0.05). DPMA, BPMA and VPMA=pea milk analogues pretreated with dry milling, blanching and vacuum, respectively, RT=retention time, RI=retention index

In this study, the main volatiles found at relatively higher concentrations (>10 µg/L) were hexanal and 2-heptanone in DPMA, 2-ethyl-furan, 1-pentanal, hexanal, 2-heptanone, 2-pentyl-furan and 1-pentanol in BPMA and 2-ethyl-furan, hexanal, 2-heptanone, 2-pentyl-furan and thujone in VPMA (Table 4). Similarly, Ma *et al*. (*8*) reported that pretreatments such as blanching and dehulling can significantly alter the content and type of volatile compounds. Most of these compounds are mainly derived from linoleic acid, the most abundant fatty acid in peas. The concentration and interaction of these compounds in the system significantly influence the sensory properties (*12*, *52*).

Several studies suggest that hexanal is a key compound associated with off-flavours and that removing this compound from the material can improve its flavour (*50*, *53*). The hexanal content of BPMA heat-treated to inactivate LOX was higher than that of the pea milk analogues subjected to other pretreatments (Table 4). This result indicates that the formation of hexanal in pea milk analogues is not solely due to LOX activity, but may also result from other reaction pathways (*48*). Even the heat treatment itself, which is used to deactivate LOX, could possibly contribute to increased hexanal formation. Lin and Blank (*54*) found that hexanal is the major odour-active volatile degradation product of heated phospholipids. Similarly, Trikusuma *et al*. (*50*) reported an increase in the amounts of hexanal, 1-pentanol, 1-octen-3-ol, 2-heptanone and 2-pentyl-furan in pea protein beverages after ultra-high-temperature treatment. Moreover, Bi *et al*. (*19*) reported that although they found a significant correlation between hexanal content and LOX activity in pea milk, they only observed a 55 % reduction in hexanal content compared to a 90 % inhibition in LOX activity.

Volatile compounds that cause the off-flavour of peas can either be present naturally in the seed or formed during processing and storage. Several molecules such as hexanal, 2-pentyl-furan, 1-hexanol, nonanal, (*E,E*)-2,4-nonadienal, (*E,E*)-2,4-decadienal have been reported to have important influence on the flavour of pea milk (*8*, *17*, *49*, *50*). In addition, certain molecules such as 3-methyl-1-butanol, 1-octen-3-ol, 1-pentanol, 3-isopropyl-2-methoxypyrazine, and (*E,E*)-2,4-heptadienal are considered responsible for beany off-flavour of peas. However, it has been shown that the flavour cannot be solely attributed to the presence of specific volatiles (*17*).

### GC-O analysis of pea milk analogues

The aroma-active compounds of pea milk analogues are listed in Table 5. A total of 29 compounds were identified using GC-O analysis, with 11 of them being present in all samples, namely 2,3-butandione (butter), hexanal (green, grass), 2-methyl-3-furanthiol (medicinal), styrene (gasoline), methional (boiled potato), 2-acetyl-1-pyrroline (popcorn, rice), 1-octen-3-one (mushroom), (*Z*)-1,5-octadien-3-one (geranium, metal), benzyl alcohol (fresh, flower), durene (dirty, oxide) and (*E*)-2-nonenal (hay) (Table 5). Most identified aroma-active compounds have been previously reported in studies on pea materials and found to belong to different groups such as aldehydes, alcohols and ketones (*49*).

**Table 5:** Aroma active compounds of the pea milk analogue determined by gas chromatography-olfactometry analysis

Aroma description	Calculated RI	Reference RI	Compound	Identification	DPMA	BPMA	VPMA
Butter	632	593	2,3-Butandione	O, RI, MS	0.65	0.75	0.65
Sulfurous	705	711	Methyl thiocyanate	O, RI, MS	0.65	-	-
Green, grass	825	801	Hexanal	O, RI, MS, STD	3.00	3.50	4.00
Sour, pungent	844	847	Isopropyl butyrate	O	0.65	-	-
Medicinal	894	868	2-Methyl-3-furanthiol	O, RI	2.25	0.40	1.00
Flower	922	-	Unknown	O	-	-	0.40
Gasoline	927	893	Styrene	O, RI	1.75	4.50	4.50
Boiled potato	934	909	Methional	O, RI, STD	1.75	1.00	2.00
Popcorn, rice	951	930	2-Acetyl-1-pyrroline	O, RI, STD	4.00	4.50	5.00
Fresh	963	1000	Methyl hexanoate	O	-	0.50	-
Rubber	999	974	2-Octanone	O, MS	-	-	2.00
Mushroom	1004	977	1-Octen-3-one	O, RI	6.50	7.00	4.00
Geranium, metal	1010	983	(*Z*)-1,5-octadien-3-one	O, RI	7.00	6.50	5.00
Fresh, flower	1031	1036	Benzyl alcohol	O, RI, MS	3.00	2.00	2.50
Dirty, oxide	1084	1087	Durene	O, RI	5.00	5.50	4.00
Fat	1089	1100	3-Nonenal	O, RI	1.50	-	-
Dirty, burnt	1123	-	Unknown	O	-	5.00	-
Dirty	1147	-	Unknown	O	-	0.75	-
Fresh	1158	-	Unknown	O	-	0.50	-
Fat	1181	1192	2-Pentylpyridine	O, RI	-	0.50	-
Cucumber	1187	1187	2-Nonanol	O	-	1.25	-
Hay	1193	1162	(E)-2-nonenal	O, RI, MS	2.00	2.50	2.00
Fat	1268	1263	Decanol	O, RI, MS	-	-	0.25
Fish market	1281	-	Unknown	O	-	1.00	-
Dirty, oxide	1350	1373	Decanoic acid	O	-	-	1.25
Fresh	1353	1329	Ethylhydroxyhexanoate	O	0.25	0.50	-
Fat	1367	1333	4-Oxodecanal	O	-	1.75	-
Sweet	1367	1350	2-Undecenal	O, RI	0.50	-	-
Hay	1374	-	Unknown	O	-	1.00	-

Results represent the olfactory intensity on a 10-point scale, where 0=none or not perceptible intensity, and 10=extremely high intensity. O=olfactory identification, RI=retention indices matched to the NIST database (*33*) and literature, STD=authentic standard compounds which were analysed under the same chromatographic conditions, MS=mass spectrometry identification. DPMA, BPMA and VPMA=pea milk analogues pretreated with dry milling, blanching and vacuum, respectively

Hexanal (green, grass), 2-acetyl-1-pyrroline (popcorn, rice), 1-octen-3-one (mushroom), (*Z*)-1,5-octadien-3-one (geranium, metal), benzyl alcohol (fresh, flower) and durene (dirty, oxide) with intensities greater than 3 were identified as the main aroma-active compounds in DPMA (Table 5). The intensities of hexanal (green, grass), styrene (gasoline) and 2-acetyl-1-pyrroline (popcorn, rice) increased, while the intensities of (*Z*)-1,5-octadien-3-one (geranium, metal) and benzyl alcohol (fresh, flower) decreased in either BPMA or VPMA compared to DPMA (control) (Table 5). Zhang *et al*. (*49*) reported that the aroma-active compounds that showed a higher intensity in olfactometric analysis of pea milk were hexanal, 1-octen-3-ol and (*E,E*)-2,4-nonadienal. Liu *et al*. (*51*) identified aroma-active compounds of hexanal, methyl hexanoate, methional and benzyl alcohol in pea protein powders (concentrates and isolates). Ebert *et al*. (*55*) found hexanal, 2-nonanol, (*E*)-2-nonenal and 2-pentyl-pyridine in pea protein isolate.

## CONCLUSIONS

The results showed that the physicochemical properties of the pea milk analogues subjected to different pretreatments were generally similar, except for yield, which was higher in the samples treated with dry milling. Vacuum treatment reduced the green and pea-like notes in the descriptive sensory analysis. Additionally, vacuum-treated pea milk analogues received higher scores for aroma, flavour and overall acceptability in the consumer acceptance test. The concentration of certain volatile compounds believed to contribute to off-flavours, such as hexanal, 1-octen-3-ol and 1-pentanol, was increased in the pea milk analogues pretreated with blanching, alkaline soaking and dehulling. Although lipoxygenase (LOX) is known for its role in the production of off-flavours, the results suggest the existence of different mechanisms, as evidenced by the highest concentration of off-flavour markers in the pea milk analogues from blanched (LOX inactivated) peas. Overall, the olfactometric intensities showed only minimal variations in the different pretreatments.

The results of the study show that the off-flavour in pea milk analogues cannot be explained by LOX activity alone. However, vacuum pretreatment proved to be an effective method for removing the off-flavour. Nevertheless, further research is needed to fully investigate the effectiveness of vacuum treatment in a more efficient and large-scale system.
